# Body weight and risk of atrial fibrillation in 7,169 patients with newly diagnosed type 2 diabetes; an observational study

**DOI:** 10.1186/s12933-014-0170-3

**Published:** 2015-01-15

**Authors:** Irene Grundvold, Johan Bodegard, Peter M Nilsson, Bodil Svennblad, Gunnar Johansson, Carl Johan Östgren, Johan Sundström

**Affiliations:** Department of Cardiology, Oslo University Hospital, Ullevaal, PB 4956, Nydalen, 0424 Oslo Norway; AstraZeneca Nordic, Södertälje, Sweden; Department of Clinical Sciences, Lund University, Skåne University Hospital, Malmö, Sweden; Uppsala Clinical Research Center, Uppsala University, Uppsala, Sweden; Department of Public health and Caring Science, Uppsala University, Uppsala, Sweden; Department of Medical and Health Sciences, Linköping University, Linköping, Sweden; Department of Medical Sciences, Uppsala University, Uppsala, Sweden

**Keywords:** Epidemiology, Atrial fibrillation, Type 2 diabetes, Weight control

## Abstract

**Background:**

Obesity, type 2 diabetes and atrial fibrillation (AF) are closely associated, but the underlying mechanisms are not fully understood. We aimed to explore associations between body mass index (BMI) or weight change with risk of AF in patients with type 2 diabetes.

**Methods:**

A total of 7,169 participations with newly diagnosed type 2 diabetes were stratified according to baseline BMI, and after a second BMI measurement within 18 months, further grouped according to relative weight change as “weight gain” (>1 BMI unit), “stable weight” (+/− 1 BMI unit) and “weight loss” (<1 BMI unit). The mean follow-up period was 4.6 years, and the risk of AF was estimated using adjusted Cox regression models.

**Results:**

Average age at diabetes diagnosis was 60 years and the patients were slightly obese (mean BMI 30.2 kg/m^2^). During follow-up, 287 patients developed incident AF, and those with overweight or obesity at baseline had 1.9-fold and 2.9-fold higher risk of AF, respectively, than those with normal BMI. The 14% of the patients with subsequent weight gain had 1.5-fold risk of AF compared with those with stable weight or weight loss.

**Conclusions:**

In patients with newly diagnosed type 2 diabetes, baseline overweight and obesity, as well as modest weight increase during the first 18 months after diagnosis, were associated with a substantially increased risk of incident AF. Patients with type 2 diabetes may benefit from efforts to prevent weight gain in order to reduce the risk of incident AF.

**Trial registration:**

ClinicalTrials.gov: NCT01121315

**Electronic supplementary material:**

The online version of this article (doi:10.1186/s12933-014-0170-3) contains supplementary material, which is available to authorized users.

## Introduction

The increasing prevalence and close co-existence of obesity, type 2 diabetes and atrial fibrillation (AF) represents a current and future health challenge [1–7]. Patients with type 2 diabetes and obesity are at high risk of cardiovascular diseases, and the additional occurrence of AF adds further to their burden of disease and increases pressure on the health care system [8]. The underlying interrelations are not fully understood, but both obesity and diabetes may cause atrial remodelling, influencing AF substrate [9–12]. In one study, the well-known obesity-AF association seemed to be mediated partially by the presence of diabetes [13]. Also after adjusting for known risk factors, including body mass index (BMI), incident AF appears more common in diabetic patients, suggesting additional effects of e.g. insulin resistance, inflammation or autonomic changes, which may act as AF mediators [14–18]. However, the importance of body weight for the AF burden in diabetes is not well characterized.

We hypothesized that higher BMI and subsequent weight gain following the diagnosis of type 2 diabetes, were associated with increased AF risk. We aimed to explore this in a large primary care based cohort of patients newly diagnosed with type 2 diabetes.

## Methods

### Study population

This was an observational cohort study based on the “Retrospective Epidemiological Study to Investigate Outcome and Mortality with Glucose Lowering Drug Treatment in Primary Care” (ROSE) study [19]. In 2010, patient data were extracted from 84 primary care centers in Sweden, representing 8% of the total centers, by the Pygargus Customized eXtraction Program. Data on all patients having a diagnosis of either type 2 diabetes and/or a prescription of glucose lowering drugs between 1999 and 2009 were extracted, as described in detail elsewhere [20,21]. Patients were excluded from the study cohort if; they had a previous history of diabetes from the medical records, were aged <35 years or >79 years, had active cancer or previous cardiovascular disease (CVD), or missing data on BMI at baseline and within 1 1/2 year after the diabetes diagnosis. They were also excluded if they developed CVD (including AF) or cancer during the time between the two BMI measurements. Patients with stable angina pectoris (AP) were allowed to participate. To minimize the risk of previously undetected AF, we included only patients that had established a first contact at their respective primary care centres minimum 15 months before baseline. Finally, 7,169 patients with newly diagnosed type 2 diabetes, but without comorbidity as mentioned above, and with regular contact with the health system, were eligible for the present analysis.

Baseline data, extracted from electronic patient charts, included systolic and diastolic blood pressure (BP), cholesterol, HbA1c and creatinine, as well as lipid lowering, glucose lowering and BP-lowering drugs. Baseline values were calculated as means of the last three measures within 15 months prior to and 14 days after the date of the diabetes diagnosis. Baseline BMI was defined as the measurement closest to the date of new-onset diabetes, restricted to the interval from 15 months prior to and within 45 days after diagnosis. BMI was analysed both in quintiles and in groups according to the World Health Organisation’s classification: normal weight (BMI 18.5-24.9), overweight (BMI 25–29.9) and obesity (BMI ≥ 30 kg/m^2^) [22].

The second or “within 1.5-year” BMI was identified in the interval between 46 days and 18 months after the diagnosis of type 2 diabetes, choosing the latest value if several measurements were available. Very few patients (4%) had less than 120 days between baseline and second BMI measurements. The mean time period between the two BMI measurements were 421 days (±120) for the weight gain group, 396 days (±134) for the stable weight group and 402 days (134) for the weight loss group. BMI change was calculated by subtracting baseline BMI from the second BMI. The participants were stratified according to a relative weight change of 1 BMI unit or more (on average ~3.6 kg in this cohort) in the following groups; weight gain (>1 BMI unit), stable weight (+/− 1 BMI unit) and weight loss (<1 BMI unit).

### Follow-up

The endpoint, incident AF, was documented by linking the participants’ unique personal identification number to validated national health registries and electronic patient records from the primary care centers. Incident AF includes both atrial fibrillation and flutter as they shared the same code number in the International Statistical Classification of Diseases system. We were unable to differentiate between paroxysmal, persistent or permanent AF.

The follow-up time started after the second BMI measurement for each patient and all were followed to the first event of AF, death or Dec 31th 2009. The study, complying with the Helsinki declaration, was approved by the Regional Ethical Review Board, Uppsala, Sweden.

### Statistical methods

Differences in baseline data were tested with Student’s t-test and Pearson Chi-square test according to data type. The risk of incident AF was estimated using Cox proportional hazard analyses. A regression spline model was used to assess the shape of the association between baseline BMI as a continuous variable, and incident AF. Two adjustment models were chosen to assess the risk of incident AF related to weight change groups – Model A: Age, gender, BMI at baseline and previous AP and model B: Model A + systolic BP. Previous AP was included due to significant differences in baseline data. In model B, missing baseline systolic BP data were imputed, which involves calculating average results over a set of imputed datasets by using Gibbs sampler and predictive mean matching. Directed acyclic graphs (DAGs) were used to minimize the risk of bias and to identify a primary adjustment model [23]. Additionally, we analysed the association between weight change and incident AF in a sub-sample of patients with BMI ≥30 kg/m^2^ at baseline. The proportional hazards assumptions were assessed by examining Schoenfeld residuals. The statistical package R, version 2.15.3, was used for all analyses. P-values and confidence intervals (CI) were two-tailed and on the 95% confidence level.

## Results

**Figure 1:**
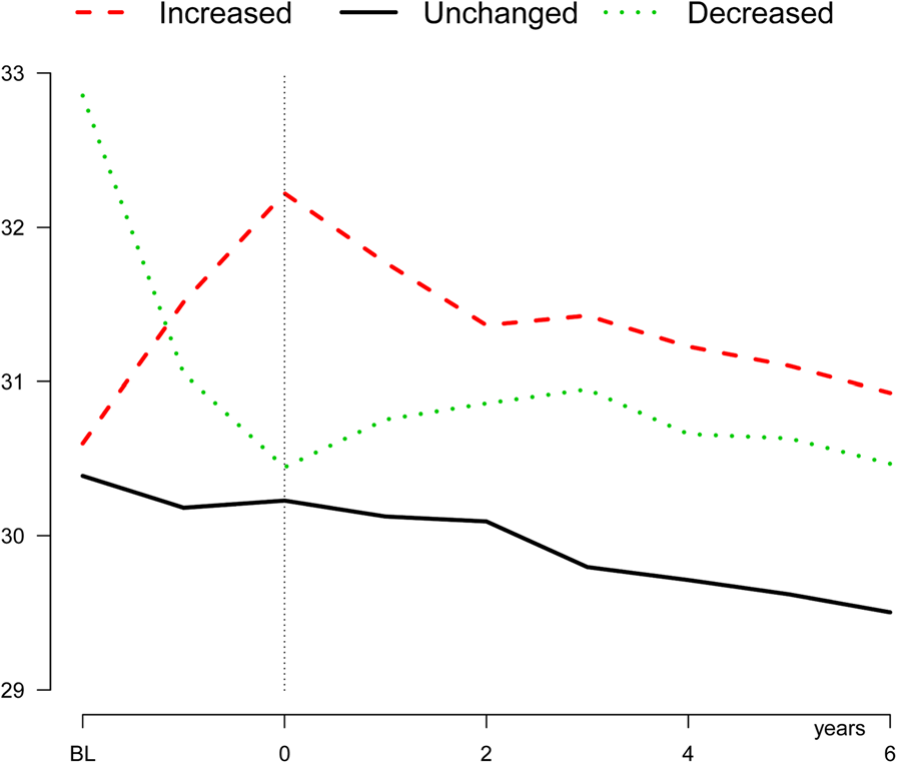
**Shows BMI at baseline and during follow-up according to weight change groups during the first 18 months after diabetes diagnosis.**

Average age was 60 years, 55% were men and mean BMI was 30.2 kg/m^2^ at baseline. Table 1 shows the baseline characteristics by weight change categories. Most patients had stable weight (52%) and more patients had a decrease (34%) than an increase (14%) in relative weight. The mean time between baseline- and second BMI was 1.3 years; hence for simplicity we have called the second measurement “within 1.5-year” BMI. Patients with weigth gain after newly diagnosed diabetes were slightly younger and had lower HbA1c levels compared to the stable weight group. Patients who lost weight were more often women with higher baseline BMI , HbA1c and BP compared to the stable group. A history of AP was less prevalent in both the weight gain and the weight loss groups compared to the stable weight group. Approximately 1/3 of the patients were initiated on at least one glucose lowering drug at the time of diabetes diagnosis. More patients were initiated on sulfonylurea and insulin and fewer on metformin in the weight gain group compared to the stable weight group (Additional file 1: Table S1). Patients were followed for up to 9 years, with median follow-up time of 4.6 years (38,300 patient-years). After the initial BMI changes, the stable weight and weight gain groups showed a steady decline in BMI, whereas the BMI trend was essentially unchanged in the weight loss group during follow-up. Both the weight gain and weight loss groups remained at higher BMI levels compared to the stable weight group, with the highest levels consistently found in the weight gain group (Figure 1).

**Table 1:** **Characteristics of patients with type 2 diabetes related to weight changes within 18 months after diagnosis**

	**Weight gain**		**Stable weight**		**Weight loss**	
	**(n = 1023)**		**(n = 3736)**		**(n = 2410)**	
Age, years	58.0	(10.3)***	60.0	(10.2)	59.3	(10.4)**
Men, n (%)	578	(56.5)	2142	(57.3)	1095	(45.4)***
BMI, kg/m^2^	30.3	(5.8)	30.3	(5.1)	32.8	(5.8)***
BMI ”1.5-year”	32.4	(6.1)***	30.2	(5.1)	30.3	(5.5)
HbA1c, %	6.5	(0.8)***	6.7	(0.8)	6.9	(0.8)***
Cholesterol, mmol/l	5.7	(1.2)	5.6	(1.1)	5.7	(1.2)
LDL, mmol/l	3.5	(1.0)	3.5	(0.9)	3.5	(1.0)
HDL, mmol/l	1.5	(0.9)	1.4	(0.8)	1.4	(0.8)
Triglycerides, mmol/l	2.3	(2.2)	2.2	(1.6)	2.3	(2.0)*
Systolic BP, mmHg	145.5	(17.9)	145.7	(17.6)	147.4	(17.5)***
Diastolic BP, mmHg	84.0	(9.3)	83.3	(9.4)	84.7	(9.2)***
Creatinine, mmol/l	78.2	(18.0)*	79.5	(17.0)	78.4	(18.7)*
Estimated GFR, ml/min	85.3	(17.5)***	83.0	(16.8)	82.5	(17.5)
Angina pectoris, n (%)	38	(3.7)*	206	(5.5)	99	(4.1)*

All numbers are means (±1 standard deviation) or numbers (percent). Significant difference from the group with stable weight: *p < 0.05, **p < 0.01, ***p < 0.001, BMI = Body mass index, LDL = Low density lipoprotein, HDL = High density lipoprotein, BP = Blood pressure, GFR = Glomerular filtration rate.

### BMI at baseline and AF risk

**Figure 2:**
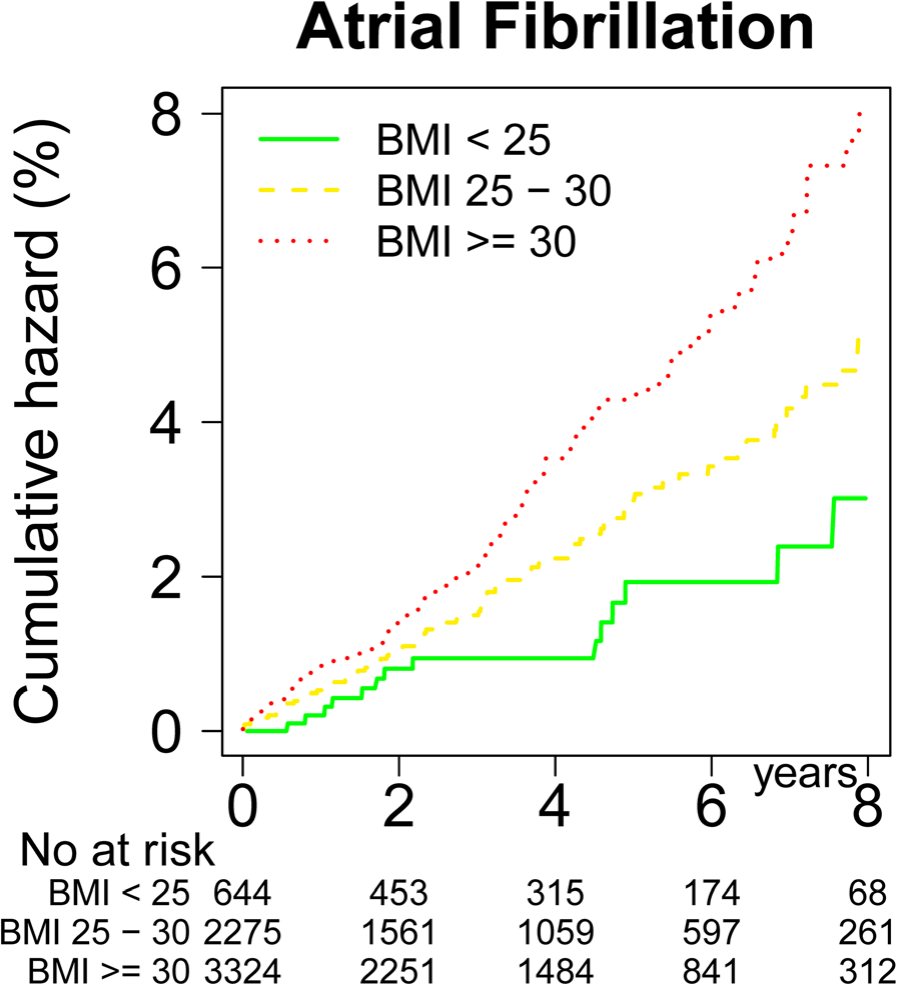
**Shows cumulative hazard of AF according to baseline BMI groups (WHO classification) adjusted for age, gender and systolic BP.**

During the follow-up period 287 patients developed incident AF. The risk of AF increased gradually with higher baseline BMI groups or BMI quintiles. When exploring the relationship between adjusted AF risk and baseline BMI using a cubic spline model, a near linear relationship was found showing increased AF risk with increasing BMI (detailed data not shown). The adjusted risk of AF was 1.9 (95% CI 1.1-3.1) and 2.9 (1.7- 4.7) for the overweight and obesity groups, respectively, compared with the patients with normal BMI (Table 2 and Figure 2). Correspondingly, the BMI quintiles showed a gradually risk increase of AF with hazard ratio 3.5 (2.3-5.4) for the highest compared to the lowest BMI quintile.

**Table 2:** **Relative risks of atrial fibrillation in hazard ratios according to baseline BMI groups and quintiles**

	**Atrial fibrillation**			
	**Number of events (%)**		**Hazard ratio (95%** **CI)**	
**Total** (n = 7169)	287	(4.0)		
**BMI groups:**				
BMI < 25 (n = 750)	17	(2.3)	1.00	Reference
BMI 25–30 (n = 2579)	100	(3.9)	*1.85*	*(1.10–3.09)*
BMI ≥ 30 (n = 3840)	170	(4.4)	*2.85*	*(1.73–4.71)*
**BMI quintiles:**				
Q1 -BMI ≤ 26.2 (n = 1278)	30	(2.3)	1.00	Reference
Q2 -BMI 26.3-28.7 (n = 1366)	56	(4.1)	*1.78*	*(1.14-2.77)*
Q3 -BMI 28.8-31.3 (n = 1486)	59	(4.0)	*1.91*	*(1.23-2.97)*
Q4 -BMI 31.4-34.8 (n = 1489)	70	(4.7)	*2.63*	*(1.71-4.04)*
Q5 -BMI ≥ 34.9 (n = 1550)	72	(4.6)	*3.52*	*(2.29-5.43)*

Adjusted for age, gender, systolic blood pressure and previous angina pectoris. CI = confidence interval, BMI = body mass index, Q = quintiles.

### Relative weight changes and AF risk

**Figure 3:**
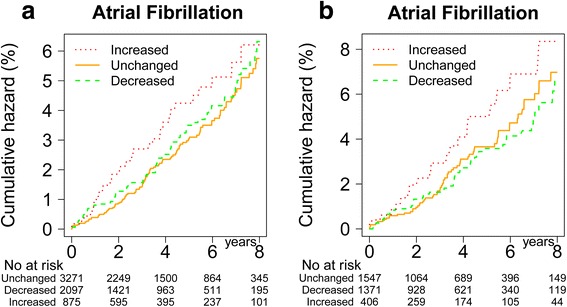
**Shows cumulative hazard of AF according to weight change groups during the first 18 months after diabetes diagnosis adjusted for age, gender, BMI at baseline, previous AP and systolic BP in a) the total cohort, and b) the subgroup of patients with baseline obesity.**

The number of patients with AF was 49 (4.8%) in the weight gain group, 139 (3.7%) in the stable group and 99 (4.1%) in the weight loss group, and the unadjusted incidence rates of AF (per 1,000 patient years) were 11.6 (8.6 – 15.1), 9.0 (7.6 – 10.6) and 10.2 (8.3 – 12.3), respectively. The risk of incident AF in adjusted analyses were 1.5-fold (1.1-2.1) higher in patients whose weight increased than in the stable weight group in model A, and almost identical in model B (Table 3). The AF risk was not different in patients with weight loss compared to those with stable weight. The adjusted cumulative incidence curves in Figure 3a) demonstrate that the increased rate of AF in the weight gain group is apparent at approximately 1 year with full separation at year 2; beyond this point in time the curves for weight gain, respectively stable weight seem parallel. In the subgroup of patients with BMI ≥ 30 kg/m^2^ at baseline (n =3,840), 170 AF events occurred during follow-up. The weight gain patients had a comparable relative risk of AF (1.6-fold, 1.0-2.5) in this obese subgroup (Table 3 and Figure 3b).

**Table 3:** **Relative risks of atrial fibrillation in hazard ratios according to weight change groups**

	**All**	**Weight gain**		**Stable weight**		**Weight loss**	
Total cohort							
-number of patients	7169	1023		3736		2410	
-number of AF events	287	49		139		99	
Model A: Hazard ratio (95% CI)		*1.54*	*(1.11–2.14)*	1.00	Reference	1.07	(0.82–1.39)
Model B: Hazard ratio (95% CI)		*1.53*	*(1.10–2.12)*	1.00	Reference	1.06	(0.81–1.38)
**Subgroup with BMI ≥ 30**							
-number of patients	3840	468		1790		1582	
-number of AF events	170	27		78		65	
Model A: Hazard ratio (95% CI)		*1.60*	*(1.03–2.49)*	1.00	Reference	0.94	(0.67–1.31)
Model B: Hazard ratio (95% CI)		*1.59*	*(1.02–2.47)*	1.00	Reference	0.93	(0.67–1.30)

Model A: Adjustments for age, gender, body mass index at baseline and previous angina pectoris. Model B: Adjustments as in Model A + systolic blood pressure; included imputation of missing blood pressure data. AF = atrial fibrillation, CI = confidence interval, BMI = body mass index.

We also explored if there was any difference between genders and found similar gradual risk increase of AF with higher baseline BMI. Generally the incidence of AF among women in our cohort was lower than among men with unadjusted incidence rates of AF in the weight gain group 14.4 (10.1 – 19.8) per 1,000 patient years in men and 8.0 (4.7 – 12.9) in women. In the Cox analyses (model B without adjustment for gender) the association between weight gain and AF risk was still significantly higher compared with the stable weight group in men, but not in women, possibly influenced by reduced statistical power (hazard ratio 1.6, 1.1-2.4 in men and 1.4, 0.8-2.5 in women).

## Discussion

In this large primary care based cohort of type 2 diabetes patients, we observed a close association between body weight – in terms of both baseline BMI and weight change during the first 18 months after diabetes diagnosis – and the subsequent development of AF. Overweight and obese patients had nearly doubled and tripled AF risk, respectively, compared to normal-weight patients. Patients with early weight gain had a 1.5-fold risk of incident AF compared to those who kept a stable weight. The AF risk was generally lower in women and the association between AF risk and weight gain only reached statistical significance in men.

AF and diabetes often co-exist [4,7,14,17]. Most studies show that diabetes is associated with a higher risk of AF, although the results have been somewhat conflicting [15,16]. Also in our study we found a high incidence rate of AF (9.7 per 1,000 patient years) during up to 9 years follow-up in more than 7,000 predominantly middle-aged type 2 diabetes patients, treated in Swedish primary care centers. The importance of body weight for the increased AF burden in diabetes is not well described, but one case–control study has indicated that the relationship between BMI and AF was stronger in patients with diabetes than in those without [13]. Accordingly, in our diabetic cohort there was a consistent and gradual risk increase of AF with increasing baseline BMI, and the associations between obesity and AF risk compared with normal BMI were of larger magnitude (hazard ratio 2.9) than previously shown in the general population (hazard ratios, obesity vs. normal BMI varying from 1.3 to 2.3 in different studies) [9,24].

In The Swedish Primary Prevention Study, weight gain from age 20 to midlife compared with stable weight was an independent long-term predictor of AF [10]. Among female healthcare professionals in the Women’s Health Study a change in weight category during the first 5 years of follow-up was associated with a slightly higher AF risk in subjects who progressed from non-obese (BMI <30 kg/m^2^) to obese weight category, than in those who were obese both at baseline and follow-up [12]. In the present study we also observed that short-term weight gain was associated with an increased AF risk, whereas weight loss did not lower the risk. To be noticed in our cohort, is that the weight loss group started with the highest baseline BMI, and after reduction their BMI was still >30 kg/m^2^. Although we made adjustments for baseline BMI, the patients with initial weight loss stabilized their weight during follow-up, while the stable weight group showed a steady decrease.

Obesity has been associated with impaired left ventricular diastolic function and left atrial dilatation, which may favour the development of AF. In the Framingham cohort the association of BMI with AF risk became insignificant after adjustments for left atrium size, leading to the conclusion that left atrium enlargement due to obesity accounted for the entire observed risk association with AF [9]. Body size, and thus obesity, is considered to be one of the most important determinants of left atrial dimension [25]. In patients with diabetes, obesity – as compared to normal weight – appears to be even more important for AF development than in the non-diabetic population. The relationship between diabetes, obesity and incident AF is not fully understood, but both diabetes and high body weight may be causally related to increased risk of AF through pathophysiological processes resulting in structural and electrical remodelling of the heart, influencing the AF substrate [3,26]. Incident AF appears more common in diabetic patients, suggesting additional effects of e.g. insulin resistance, inflammation or autonomic changes, which may act as AF mediators. Insulin resistance in type 2 diabetes – also a key characteristic of obesity – is associated with hypertension and left ventricular hypertrophy. Furthermore, both obesity and diabetes are associated with underlying autonomic disturbances that may also influence atrial arrhythmogenicity, thereby facilitating local AF triggers [14,15].

### Strengths and limitations

The strengths of the ROSE study include the large number of patients with newly diagnosed type 2 diabetes in a genuine clinical setting in primary health care, and the mean 4.6-year follow-up period for endpoint measurements recorded in nationwide health care registries. Limitations of the study include missing data on diet, waist circumference and physical activity in our database. The baseline demographics show more common use of sulfonylurea and insulin in the weight gain group. Regretfully, we did not have statistical power to explore the potential influence of each glucose lowering drug separately, which might be relevant as some drugs (ie. metformin) are reported to slightly reduce weight and others (sulfonylurea and insulin) to increase weight [27]. A very recent study found that among patients with type 2 diabetes in Taiwan, metformin use was associated with a decreased risk of AF [28]. Antihypertensive medication during follow-up might also influence the risk of incident AF independently of weight change [29].

We did not perform analysis with adjustments for the competing risk of death. Previous results from the same cohort has shown that weight gain increases CVD mortality, indicating that mortality may be a competing risk in the analyses of AF incidence; hence the weight gain-AF association may be even higher than those observed in the present study [20].

## Conclusion and clinical implications

In primary care patients with newly diagnosed type 2 diabetes, the overweight and obese patients had nearly doubled and tripled risk of AF, respectively, compared to the normal-weight patients. Additionally, a modest weight gain during the first 18 months after diabetes diagnosis was associated with an increased risk of incident AF. It is possible that prevention of further weight gain, especially among already obese persons with newly diagnosed type 2 diabetes, is particularly important to prevent increased AF risk. This finding might also influence the choice of glucose lowering medication as some drugs are reported to have a beneficial impact on weight.

### Ethical Approval

The study, which complied with the declaration of Helsinki, was approved by the Regional Ethical Review Board in Uppsala, Sweden

## Additional file

Additional file 1: Table S1. Drug treatment of patients with newly diagnosed type 2 diabetes according to weight change categories.

## Abbreviations

AF: Atrial fibrillation
BMI: Body mass index
ROSE: Retrospective Epidemiological Study to Investigate Outcome and Mortality with Glucose Lowering Drug Treatment in Primary Care”
CVD: Cardiovascular disease
AP: Angina pectoris
BP: Blood pressure
DAGs: Directed acyclic graphs
CI: Confidence intervals
