# Supplementary material for: Body weight and risk of atrial fibrillation in 7,169 patients with newly diagnosed type 2 diabetes; an observational study
Source: Cardiovasc Diabetol. 2015 Jan 15;14:5. doi: 10.1186/s12933-014-0170-3 (PMC4299152; doi:10.1186/s12933-014-0170-3)
Supplement: Additional file 1: Table S1. — Drug treatment of patients with newly diagnosed type 2 diabetes according to weight change categories. [file 12933_2014_170_MOESM1_ESM.doc]

|  | Weight gain (n = 1023) | | Stable weight (n = 3736) | | Weight loss (n = 2410) | |
| --- | --- | --- | --- | --- | --- | --- |
| Glucose lowering drug, n (%) | 319 | (31.2) | 1091 | (29.2) | 7106 | (29.5) |
| - Metformin, n (%) | 167 | (16.3)* | 731 | (19.6) | 601 | (24.9)*** |
| - Sulfonylurea, n (%) | 121 | (11.8)* | 344 | (9.2) | 105 | (4.4)*** |
| - Insulin, n (%) | 32 | (3.1)*** | 49 | (1.3) | 17 | (0.7)* |
| - Other OGLD, n (%) | 17 | (1.7) | 36 | (1.0) | 15 | (0.6) |
| Low dose ASA, n (%) | 104 | (10.2)* | 479 | (12.8) | 250 | (10.4)** |
| Statins, n (%) | 178 | (17.4)* | 769 | (20.6) | 441 | (18.3)* |
| - Simvastatin, n (%) | 136 | (13.3)* | 600 | (16.1) | 349 | (14.5) |
| - Atorvastatin, n (%) | 26 | (2.5) | 121 | (3.2) | 70 | (2.9) |
| - Rosuvastatin, n (%) | 2 | (0.2) | 11 | (0.3) | 2 | (0.1) |
| Antihypertensives, n (%) | 387 | (37.8)** | 1607 | (43.0) | 1076 | (44.6) |
| - ACE, n (%) | 135 | (13.2) | 562 | (14.6) | 372 | (15.4) |
| - ARB, n (%) | 90 | (8.8) | 372 | (10.0) | 240 | (10.0) |
| - CCB, n (%) | 104 | (10.2) | 462 | (12.4) | 298 | (12.4) |
| - Thiazides, n (%) | 87 | (8.5) | 380 | (10.2) | 302 | (12.5)** |
| - Alphablockers, n (%) | 7 | (0.7) | 21 | (0.6) | 11 | (0.5) |
| - Betablockers, n (%) | 217 | (21.2) | 855 | (22.9) | 553 | (22.9) |
| Weight reducing drugs, n (%) | 20 | (2.0) | 51 | (1.4) | 52 | (2.2)* |
| Anti-depressive drugs, n (%) | 84 | (8.2) | 255 | (6.8) | 183 | (7.6) |
| Benzodiazepines, n (%) | 6 | (0.6) | 25 | (0.7) | 13 | (0.5) |

Significant difference from the group with stable weight: * = p<0.05, ** = p<0.01, *** = p<0.001. OGLD=Oral glucose lowering drugs, ASA=Acetylsalicylic acid, ACE=Angiotensin converting enzyme inhibitor, ARB=Angiotensin receptor blocker, CCB=Calcium channel blocker

**Additional file 1: Table S1** **Drug treatment of patients with newly diagnosed type 2 diabetes**
